# Environmental factors on the probability of pregnancy in early or conventionally weaned beef cows

**DOI:** 10.1590/1984-3143-AR2023-0054

**Published:** 2023-09-18

**Authors:** Ricardo Zambarda Vaz, José Fernando Piva Lobato, Javier Alexander Bethancourt-Garcia, Rangel Fernandes Pacheco, Nathália Pasi Reis, Dayana Bernardi Sarzi Sartori, Sabrina Amália Jappe, João Restle

**Affiliations:** 1 Universidade Federal de Santa Maria, Palmeira das Missões, RS, Brasil; 2 Universidade Federal do Rio Grande do Sul, Porto Alegre, RS, Brasil; 3 Universidade Federal de Lavras, Lavras, MG, Brasil; 4 Instituto Federal Farroupilha, Frederico Westphalen, RS, Brasil; 5 Universidade Federal de Santa Maria, Santa Maria, RS, Brasil; 6 Universidade Federal de Goiás, Goiânia, GO, Brasil

**Keywords:** ambience, calving weight, Julian calving date, lactation time, postpartum weight gain

## Abstract

Potential variables for the reproductive success of beef cows were evaluated. Included in the model were the age of the cow at calving; the interval between the Julian calving date and the end of the breeding season; the body weight and conditions at calving, at 75 days post-partum and at the end of the breeding season; and the mean daily variation in weight between these dates. Logistic regression was used in the analysis, with the parameters evaluated using the odds ratio statistic, estimating the chance of pregnancy. The mean rate of pregnancy was 84% and 55% for early and late-weaned cows, respectively. For early weaned cows, the regression variables were the Julian calving date, age of the cow, weight gain from calving to 75 days post-partum, and from 75 days post-partum to the end of the reproductive period. For late-weaned cows, there were only two regression variables, weight at calving and weight gain from calving to the end of the reproductive period. For every year above the average age of the herd, early weaned cows have an 80.9% greater chance of pregnancy, while a reduction of one year reduces the chance of pregnancy by 44.7%. In early weaned cows, every seven days after the mean Julian calving date reduces the chances of pregnancy by 22.6%, whereas every seven days before the mean calving date increases pregnancy by 29.2%. Greater gains in cow body weight, from calving to the end of the reproductive period, determine a greater probability of pregnancy.

## Introduction

In assessing the bioeconomics of breeding systems, reproductive variables are the most representative in terms of costs and successful responses (Pravia et al., 2014). The search to increase production, requiring the cow to gestate and wean one calf per year under limiting environmental conditions, results in herds with low rates of reproduction. Competition with grain production for prime areas of land in terms of soil and fertility, is leaving livestock systems with non-ideal production environments and conditions (Alforma et al., 2023).

Adverse environmental conditions together with high nutritional requirements during the post-partum period of beef cows cause a negative energy balance (Diskin et al., 2003; Vaz and Lobato, 2010), which determines a halt in reproductive activities to maintain milk production (Montiel and Ahuja, 2005). Discontinuing cows from milk production through early weaning is one way of recovering their body condition and improve the rate of pregnancy under restricted feeding (Vaz et al., 2010). The process of transforming pasture into milk, and from milk into kilograms of calf is inefficient, as cows and calves when fed separately, show 43% greater efficiency in converting total digestible nutrients into body weight (Martins et al., 2012).

The early weaning of calves up to 80 days post-partum, together with a mean gestation period of 285 days, ends the productive year of the cows. Early weaning affords significant gains in physiological and productive variables (Vaz and Lobato, 2010; Ungerfeld et al., 2011), especially under nutritional conditions that are inadequate for simultaneous maintenance, lactation and pregnancy (Orihuela and Galina, 2019). Variables such as weight, weight gain, body condition score and the interval from calving to the end of the reproductive period (Pacheco et al., 2022), and lactation (Camargo et al., 2022) affect the reproductive performance of beef cows. If early weaning improves the reproductive efficiency of the herd (Vaz et al., 2010), in which variables does it interfere, and to what extent do these affect the reproductive performance of the cows?

The aim of the study was to evaluate environmental effects by means of developmental and performance characteristics, and identify in which way they affect the pregnancy of early or conventionally weaned beef cows.

## Methods

### Location and animals

The experiment was carried out at the Granja Itú beef cattle ranch, in the district of Itaqui, Rio Grande do Sul (29°12' S, 55°36' W). The soil is classified as a dystrophic Red Latosol. According to the Köppen classification, the climate is subtropical (Alvares et al., 2013). All animal handling and procedures were approved by the Ethics Committee for Animal Use (CEUA) of the “Universidade Federal de Santa Maria” under number 2388280122.

We evaluated 283 lactations in Braford beef cows with ages ranging from three to five years and distributed in two weaning ages at 76 days (175 lactations) and at 148 days (109 lactations), always being managed in a single group. During pregnancy, the cows were kept on natural pasture at an animal load of 320 kg live weight/ha; during the post-partum period they were kept on a pasture of *Braquiaria Brizanta cv* ‘Marandu’ at an animal load of 800 kg live weight/ha, until the conventional weaning date.

Calving occurred over a period of 70 days, with the cows uniformly distributed by calving date and early or conventional weaning age. The cows and their calves were weighed during the first 24 hours after calving, at the early and conventional weaning age, and at the start and end of the reproductive period. The average daily weight gain (ADG) was determined by the weight difference between each weighing divided by their interval in days between them. The body condition score (BCS) was evaluated during weighing (Lowman et al., 1976), and given a value from 1 to 5, where 1 = very thin and 5 = very fat.

Early weaning took place from December to January, as the calves reached between 68 and 80 days of age. Conventional weaning took place on a single date, 30 days after the end of the reproductive period.

Reproduction was by natural breeding at a bull/cow ratio of 1:25 and lasted 63 days. The bulls were previously assessed for libido and by andrological examination. Pregnancy was diagnosed by ultrasound 30 days after the end of the reproductive period.

### Tested variables

The variables under test were the age of the calf at weaning; the interval between calving and the end of the reproductive period; the body weight of the cow at calving, at 75 days post-partum and at the end of the reproductive period; the body condition score (BCS), at calving, 75 days post-partum and at the end of the reproductive period; the ADG from calving to 75 days post-partum, and from 75 days post-partum to the end of the reproductive period.

### Statistical analysis

The SAS statistical package (Statistical Analysis System, v 9.2) was used for the statistical analysis. The response variable for rate of pregnancy was given a value of one for pregnant females and zero for non-pregnant females, and analysed by logistic regression using the LOGISTIC procedure. Multicollinearity between the predictor variables was diagnosed by analysing the Pearson correlation matrix and measuring the variance inflation factor, condition index, eigenvalues (λ) and the proportion of variance associated with each eigenvalue (Khalaf and Iguernane, 2016). From this analysis, it was possible to select the set of covariables to be used to construct the models, using the significance of each covariable obtained in the likelihood ratio test. To do this, several multiple regression models with linear, and with linear and quadratic effects, as well as their interactions, were tested using the stepwise method. The probability limit for both inclusion and remaining in the model was 0.25 and 0.30 respectively (Hosmer et al., 2013). The choice of the best model to be adopted considered the Hosmer and Lemeshow test for goodness of fit, with *p*>0.30 (Hosmer et al., 2013).

After adjusting the model (estimating the *βi* parameters), the significance of the variables resulting from the model was tested in order to determine whether the independent variables were significantly related to the probability of calving. To assess the quality of the adjusted model and the individual significance of the set of parameters used in the model, the Wald test and the Score test were used.

The multiple regression model (Model I - early and Model II - conventional) adjusted for the calving rate of the early weaned or conventionally weaned cows is expressed by the following equations:


$$Pi=\;\frac{exp(yi)}{1+exp(yi)}={\left[1+\exp\left(-Yi\right)\right]}^{-1}$$ (1)

where P_i_ in Model I is a pregnant diagnosis for the i-th cow under early weaning:


yi=µ+β1X1i+β2X2i+β3X3i+β4X4i+εi (2)

where µ is a constant; X1i, age at calving of the i-th cow; X2i, Julian calving date of the i-th cow; X3i, ADG from calving to 75 days post-partum of the i-th cow; X4i, ADG from 75 days post-partum to the end of the reproductive period for the i-th cow; εi, random error associated with the i-th cow.

P_i_ in model II is a pregnant diagnosis for the i-th cow under conventional weaning:


yi=µ+β1X1i+β2X2i+β3X3i+εi (3)

where µ is a constant; X1i, BW at calving of the i-th cow; X2i, ADG from calving to the end of the reproductive period of the i-th cow; X3i, ADG from calving to weaning i-th cow; εi, random error associated with the i-th cow.

To interpretate the coefficients, the odds ratio estimated by OR = *exp*(bk) was used, considered the ratio between two possible results, i.e. between the success (*πj*) or failure (*1-πj*) of the pregnancy. The odds ratios were based on the mean denominator of the data set for each model. The units of change in the regressor variables were: one year for the age at calving; 10 kg for the BW at calving; seven days for the Julian calving date; 0.100 kg for the ADG from calving until 75 days post-partum; 0.100 kg for the ADG from 75 days post-partum until the end of the reproductive period; and 0.100 kg for the ADG from calving until the end of the reproductive period.

## Results

Descriptive statistics show similarity in mean age and Julian calving date between the early weaned and t conventionally weaned cows (Table 1). The performance characteristics of weight, weight gain and body condition score differ as a result of the different lactation periods the cows underwent, with an average pregnancy of 84% and 55% for the early and conventionally weaned cows, respectively. The Julian calving dates of 289 and 282 days represent the 17th and 10th of October for early weaned and conventionally weaned cows, respectively.

**Table 1:** Descriptive analysis of data from cows submitted to the early or conventional weaning of their calves.

	**Early**			**Conventional**		
	**N**	**Mean Value**	**SD**	**N**	**Mean Value**	**SD**
Age, years	175	3.8	0.8	109	3.6	0.8
BW, kg						
Calving	175	359.6	61.0	109	337.2	45.5
75 days post-partum	175	379.8	56.0	109	359.1	48.6
End of reproductive period	175	399.9	56.8	109	370.2	51.6
BCS, points						
Calving	175	2.9	0.69	109	2.7	0.6
75 days post-partum	175	3.2	0.54	109	3.1	0.5
End of reproductive period	175	3.6	0.57	109	3.2	0.5
ADG, kg of BW/day						
C-75d	175	0.281	0.297	109	0.264	0.267
75d-TRP	175	0.300	0.241	109	0.165	0.236
CRP	175	0.300	0.189	109	0.249	0.144
JCD, days	175	289	20.5	109	282	20.9
Calving rate, %	175	84.0	3.6	109	55.0	4.9

BW: body weight; BCS: body condition score; ADG: average daily gain; C-75d: from calving to 75 days post-partum; 75d-TRP: from 75 days to the end of the reproductive period; CRP: from calving to the end of the reproductive period; JCD: Julian calving date.

Two equations adjusted for the intercept were created after verifying the diagnosis of multicollinearity, one for cows weaned early and the other for conventionally weaned cows (Table 2). The regression equations for both groups of cows were built using the significant variables only (Table 3). In Model I, for early weaned cows, the following variables explain the probability of pregnancy: the age of the cow, the Julian date of calving, the ADGs from calving to 75 days post-partum, and from 75 days post-partum to the end of the reproductive period. For conventionally weaned cows, Model II includes the following variables to explain the probability of pregnancy: the effect of the Julian calving date, of the body weight at calving, and of the ADG from calving to the end of the reproductive period. It was not necessary to make any adjustments to reduce multicollinearity, as confirmed by the Hosmer and Lemeshow (HLT) statistic, with values of P=0.7955 and 0.5581 for early weaned and conventionally weaned cows, respectively (Table 3).

**Table 2:** Diagnosis of multicollinearity between the coefficients included in early or conventional weaning.

**Number**	**VIF**	**Λ**	**CI**	**Proportion of variance decomposition associated with each eigenvalue**			
				**Age**	**JCD**	**ADG**	
**Early**						**C-75d**	**75d-TRP**
1	0	4.1788	1.0000	0.0010	0.0001	0.0178	0.0140
2	2.2259	0.4749	2.9665	0.0010	0.0001	0.8941	0.0896
3	2.3624	0.3224	3.6002	0.0059	0.0006	0.0597	0.7478
4	1.0124	0.0226	13.5956	0.5196	0.0065	0.0262	0.0063
5	1.1432	0.0013	57.5247	0.4724	0.9927	0.0022	0.1423
Conventional				ADG-CRP		BW-calving	
1	0	3.7948	1.0000	0.0144		0.0001	
2	1.3873	0.1931	4.4325	0.9806		0.0049	
3	1.0243	0.0096	19.8680	0.0001		0.8589	
4	1.3745	0.0024	39.5080	0.0049		0.1352	

VIF: variance inflation factor; λ: eigenvalue; CI: condition index; ADG: average daily gain; C-75d: from calving to 75 days post-partum; 75d-TRP: from 75 days to the end of the reproductive period; CRP: from calving to the end of the reproductive period; JCD: Julian calving date.

**Table 3:** Regressor variable on the rate of pregnancy and confidence limits in early or conventional.

**Early**	**Estimate**	**Standard Error**	**95% CL**	***P-value***	**HLT**
Intercept	9.4196	3.6970	2.1737 to 16.6656	0.0108	0.7985
Age	0.5925	0.4177	-0.2262 to 1.4112	0.1560	
JCD	-0.0366	0.0158	-0.0675 to -0.0058	0.0201	
ADG					
C-75d	1.1648	0.7359	-0.2776 to 2.6073	0.1135	
75d-TRP	1.3245	1.0811	-0.7944 to 3.4433	0.2205	
Conventional					
Intercept	-9.0638	2.2831	-13.5386 to -45890	<0.0001	0.5581
BW-calving	0.0257	0.0684	0.0123 to 0.0391	0.0002	
ADG					
CRP	3.0572	1.4718	0.1726 to 5.9419	0.0378	

CL: confidence limits; HLT: Hosmer and Lemeshow test; ADG: average daily gain; C-75d: from calving to 75 days post-partum; 75d-TRP: from 75 days to the end of the reproductive period; CRP: from calving to the end of the reproductive period; JCD: Julian calving date; BW: body weight.

There are more alternatives when working with early weaned cows in production systems, which offer the possibility of increasing the pregnancy rate of the herds. From the odds ratio statistic, for each year above the average age of the herd, early weaned cows have an 80.9% greater chance of pregnancy. However, for each year the age of the cow is reduced, the chance of pregnancy is reduced by 44.7% (Table 4).

**Table 4:** Estimate of the chances of each regressor variable on the rate of pregnancy in early or conventional weaning.

**Early**	**PE**	**95% CL**	**Increase**		**Reduction**	
			**Unit**	**Estimate**	**Unit**	**Estimate**
Age	1.809	0.798 to 4.101	1 year	1.809	-1 year	0.553
JCD	0.964	0.935 to 0.994	7 days	0.774	- 7 days	1.292
ADG						
C-75d	3.205	0.758 to 13.562	0.100 kg	1.124	-0.100 kg	0.890
75d-TRP	3.760	0.452 to 31.290	0.100 kg	1.142	-0.100 kg	0.876
Conventional						
BW-calving	0.0257	0.0123 to 0.0391	10 kg	1.293	-10 kg	0.773
ADG						
CRP	3.0572	0.1726 to 5.9419	0.100 kg	1.358	-0.100 kg	0.737

PE: point estimate; CL: confidence limits; ADG: average daily gain; C-75d: from calving until 75 days post-partum; 75d-TRP: from 75 days to the end of the reproductive period; CRP: from calving to the end of the reproductive period; JCD: Julian calving date.

In early weaned cows with births during the calving season, for every seven days after the average calving date (17 October) (JCD) the chances of pregnancy decrease by 22.6%, while for every seven days before the average calving date, the chances of pregnancy increase by 29.2%.

From calving to the end of the reproductive period, pregnancy was influenced by weight gain in the cows, regardless of the lactation period; however, in early weaned cows, performance up to weaning, and from weaning to the end of the reproductive period, had an isolated effect on pregnancy. From an average daily gain of 0.281 and 0.300 kg onwards for the pre-weaning period (from calving to 75 days post-calving) and post-weaning period (75 days post-calving to the end of the reproductive period), an increase of 0.100 kg increased the probability of pregnancy by 12.4% and 14.2%, respectively. In contrast, a reduction of 0.100 kg in the average daily gain before and after weaning determined a respective reduction of 11.0% and 12.4% in the chances of pregnancy in early weaned cows.

Post-partum performance was significant for the chances of pregnancy in conventionally weaned cows. A gain in body weight from calving to the end of the reproductive period determined a greater probability of pregnancy. From a daily gain of 0.249 kg in body weight onwards, an increase of 0.100 kg in average daily gain afforded an increase of 35.8% in the probability of pregnancy, and a decrease of 0.100 kg, afforded a decrease of 26.3%.

Body weight at calving also helps explain pregnancy in cows with a prolonged lactation period. From an average weight of 337 kg onwards, a 10 kg increase in body weight at calving in conventionally weaned cows increases the chance of pregnancy by 29.3%; on the other hand, a 10 kg reduction in body weight at calving reduces the chance of pregnancy by 22.7%.

## Discussion

Pregnancy in beef cows is influenced by various factors, mainly those related to nutrition (Cooke et al., 2021). When management conditions are not ideal, especially those related to nutrition as in the present study, all possible variables that increase pregnancy need to be correctly measured. With the correct measurements, it is possible to make use of the best alternatives for enhancing reproduction within each production system. The analyses carried out in the present study show that early weaned cows have more options for generating better reproductive results. In cows that are weaned early, after the average age and Julian calving date, post-partum weight gains determine a better reproductive result. For reproductive success, cows that suckle their calves for a longer period need to calve at a good weight, and gain weight from calving to the end of the reproductive period.

The greater flexibility in achieving positive results in the reproduction of early weaned cows is due to their lower nutritional requirement, as nutrients are not needed for milk production and can be used for weight gain and the accumulation of body reserves (Vaz and Lobato, 2010). With the lower requirement of early weaned cows (Moura et al., 2014), reproduction can be improved by an increase in weight until early weaning, or by greater weight gains during the reproductive period, affording breeding systems various alternatives, which can be managed with a view to greater profitability (Alforma et al., 2023).

The greater success of pregnancy in older bovine females, verified in the batch of early weaned cows, is consistent with reports in the literature (Vieira et al., 2005; Bitencourt et al., 2020). Young cows have a more demanding physiology, with difficulty in meeting the requirements of maintenance, lactation, reproduction and continued growth, especially under non-ideal nutritional conditions, such as those of the present study. The 80.9% superiority is probably due to the low average age (3.8 years) of the herd under study. Pacheco et al. (2022) also found an increase in pregnancy in Charolais and Nellore cows and their crosses, with a 36.6% greater chance of pregnancy for each additional year in the age of the cows. The fewer instances of pregnancy are due to the average age of the herd (6 years) studied by Pacheco et al. (2022), with the herd, in general, having already passed the growth phase. The results of the present study, together with the literature, confirm that the age of the cows is fundamental to the pregnancy of a herd. Early weaning has proved to be an interesting alternative for use in young cows, affording satisfactory results for pregnancy with each increase or decrease in the average age of the herd. The combined effects of growth and lactation in young cows have no positive correlation with reproduction (Pacheco et al., 2020; Camargo et al., 2022).

An early calving date during the calving season determines greater development and performance in the cows and their calves (Vaz et al., 2020a, b; Pacheco et al., 2022). Late-calving cows have less time for their body and physiology to recover before the next reproductive period. This increases the interval between births, as the return of post-partum ovarian activity and the subsequent manifestation of oestrus, requires uterine involution to occur (Díaz et al., 2017).

Under natural pasture and a subtropical climate as in the present study, because the calving season is in the spring, the later the birth, the greater the weight and body condition scores of the cows at calving (Vaz et al., 2020b) due to the nutritional improvement that occurs at the end of pregnancy. However, productive variables that express pre-partum performance are less significant for the probability of pregnancy than are variables of post-partum performance (Pacheco et al., 2022). On the other hand, late-calving cows lactate when the nutritional environment is inadequate and, at the same time experience the peak of lactation when already subjected to further reproduction.

Early calving cows, despite lower weight and body condition, benefit from the improvement in forage quality after calving, which ensures better nutritional levels, and are subjected to further reproduction after the peak of lactation. For beef cows, reproduction after lactation affords better body condition and greater weight gain during the reproductive period, improving reproductive performance (Shoup et al., 2015).

In the present study, the calving date had no influence on pregnancy in cows that suckle their calves for a longer period, showing the cows to be dependent on a greater calving weight and better post-partum development for reproductive success (Alforma et al., 2023). This demonstrates that the wear on the cow caused by lactation makes reproduction more difficult, since to become pregnant, cows need to give birth under favourable conditions and show adequate weight gain afterwaed. Early weaning at 60-70 days increases the chances of early pregnancy during the next reproductive period, which favours further reproduction (Vaz et al., 2010; Alforma et al., 2023), which should not harm the development of calves (Vaz et al., 2010).

Weight gain during the post-partum period determined the reproductive success of both groups of cows as a function of the weaning age of the calves. Greater variation in weight gain occurred in early weaned cows than in those weaned later. In the early weaned cows, pre- and/or post-weaning weight gains had an influence on the probability of pregnancy, while in cows that suckled for a longer period, weight gains from calving to the end of the reproductive period proved to be influential. The greater weight gains of early weaned cows are due to the lower requirements of production once lactation ceases (Vaz and Lobato, 2010; Alforma et al., 2023).

With less required for production, the nutrients ingested by the cows are directed to the accumulation of body reserves, which are necessary for reproductive success (Silveira et al., 2014). Lactation represents a substantial reorganization in the hierarchy of nutrient partitioning. Feed is consumed and digested products are assimilated and partitioned in a process governed by a physiological echelon; meeting maintenance requirements is top priority and secondary uses of absorbed nutrients are for productive functions such as milk synthesis or fetal development (Baumgard et al., 2017).

For late-weaned cows, greater body weight at calving determined a higher chance of pregnancy. This underlines that suckling is exhausting, and has an inhibitory effect on reproduction in beef cows (Vaz and Lobato, 2010). Cows that suckle their calves during the reproductive period need to have good muscle and fat reserves at calving so that, through mobilisation, they meet the negative energy balance that originated during the post-partum period, and reach the reproductive period able to conceive (Alforma et al., 2023). It should be noted that the weight at calving is important, provided that this is due to a greater deposition of muscle and fat tissue in the cow, and not to an increase in the size or body structure of the animal (Vaz et al., 2022a, b). Larger body structures determine low reproductive performance when the genotype does not adapt to the environmental conditions (Farias et al., 2018). Furthermore, when the category is primiparous cows, and the nutritional conditions are inadequate, reproductive failure is certain (Vaz et al., 2020c), and weaning the calves is advisable (Camargo et al., 2022).

It is up to system managers to choose the easiest and most adaptable of these for an increase in pregnancy in each system. Several ways can determine greater pregnancy in beef cows. Health care, mineralization of herds, ambience, interrupted weaning or even hormonal induction, but all these technologies are palliative, not solving the cow's problem, which is the lack of food. However, when situations are unfavourable to the pregnancy of beef cows, early weaning serves as a management corrective, as it affords the cows the same food supply with the conditions for storing body reserves, enhancing several variables for a positive result for the pregnancy.

## Conclusions

Cows that suckle for an average period of 148 days post-partum show a lower rate of reproduction. The presence of a suckling calf determines that for reproductive success the cow should calve showing good body weight, and perform well during the post-partum period. With the calf removed due to early weaning, post-partum performance, although significant, has less impact on the chance of pregnancy in the beef cow, but allows variables such as the age of the cow and the Julian date of delivery to have an impact on the probability of pregnancy.

## Data Availability

The datasets generated during and/or analyzed during the current study are available from the corresponding author on reasonable request, with all research data being published.
